# Evaluation of Redox-Mediated Responses of Coral Symbiotic Dinoflagellates to Nano-Selenium

**DOI:** 10.3390/md24080265

**Published:** 2026-07-31

**Authors:** Xinyu Shan, Yunting Wang, Wenxin Wang, Mingxuan Wang, Shuangqi Yue, Fengyue Qin, Menglu Dong, Waqas Ahmed, Ling Li, Senjie Lin, Sajid Mehmood, Weidong Li

**Affiliations:** 1 International Joint Research Center for Coral Reef Ecology of Hainan Province, School of Ecology, Hainan University, Haikou 570100, China; 2 School of Ecology, Hainan University, Haikou 570100, China; 3 College of the Environment & Ecology, Xiamen University, Xiamen 361102, China; 4 State Key Laboratory of Marine Environmental Science, College of Ocean and Earth Sciences, Xiamen University, Xiamen 361102, China; 5 Department of Marine Sciences, University of Connecticut, Groton, CT 06340, USA

**Keywords:** green synthesis, selenium nanoparticles, symbiotic dinoflagellates, redox regulation

## Abstract

This study investigated species-specific physiological and redox responses of two coral symbiotic dinoflagellates, *Cladocopium* sp. and *Durusdinium* sp., to green-synthesized nano-selenium (SeNP) gradients, with implications for eco-friendly marine antifouling. Growth, photosynthetic pigments, antioxidant enzymes (SOD, POD, CAT), lipid peroxidation (MDA), and osmo-protectants were assessed to elucidate mechanisms. Both species exhibited a biphasic (hormetic) response, with stimulation at low concentrations and inhibition at high levels. At 50–100 mg L^−1^, *Cladocopium* sp. showed enhanced growth, pigments, antioxidant activity, and osmotic regulation, with reduced oxidative stress, indicating improved redox homeostasis. In contrast, ≥150 mg L^−1^ disrupted redox balance and suppressed growth. *Durusdinium* sp. displayed slower growth but maintained stable pigments, consistent antioxidant activity, and low MDA, reflecting a tolerance-oriented strategy. Overall, SeNPs synergistically regulate antioxidant systems and osmotic homeostasis to balance the intracellular redox status of symbiotic dinoflagellates, indicating their potential as antioxidant agents to improve the growth performance of symbiotic dinoflagellates in coral nursery cultivation.

## 1. Introduction

Coral reefs are among the most biodiverse ecosystems on Earth, providing essential ecosystem services that sustain nearly 10% of the global population and support multi-billion-dollar industries, including tourism and fisheries [1,2]. However, long-term ocean warming, overexploitation, and increasing anthropogenic pressures have led to widespread coral bleaching and mortality, necessitating a reassessment of conservation priorities for global reef health [2,3]. Coral bleaching, primarily driven by thermal stress and other environmental factors, occurs when corals lose their endosymbiotic algae, ultimately compromising their survival [4]. Prolonged exposure to elevated temperatures and high solar irradiance disrupts the coral-algal symbiosis, leading to reduced physiological performance and, in severe cases, host mortality [5]. Therefore, the stability of the coral-dinoflagellate symbiosis is fundamental to coral health and reef persistence [6]. Coral bleaching is characterized by the loss of symbiotic dinoflagellates or their photosynthetic pigments from coral tissues, which reduces the transfer of photosynthetically fixed carbon to the host [7]. Because these symbionts make a major contribution to coral energy metabolism, factors that suppress their growth or impair their physiological performance may reduce symbiont fitness and potentially destabilize the coral-algal association. Accordingly, we evaluated the growth and physiological responses of *Cladocopium* sp. and *Durusdinium* sp. as indicators of SeNP-induced stress. However, responses observed in free-living cultures should not be interpreted as direct evidence of bleaching or impaired symbiosis in intact corals.

Nanomaterials with redox-modulating properties have attracted increasing interest because they can influence oxidative balance in a concentration-dependent manner. Among them, selenium nanoparticles (SeNPs) are of particular interest owing to their high surface reactivity, enhanced bioavailability, and potential to act as either antioxidants or pro-oxidants depending on the exposure dose [8,9,10]. Nevertheless, their effects on coral symbiotic dinoflagellates remain poorly understood. Investigating how SeNPs influence symbiont growth, photosynthetic performance, antioxidant defense, and osmotic regulation is therefore important for establishing a physiological and ecotoxicological basis for their possible future use in controlled coral nursery systems.

Symbiosis represents a long-term and intimate association between different species, often involving endosymbiosis, where one organism resides within the cells of another [11]. Members of the family Symbiodiniaceae are among the most successful micro-eukaryotic symbionts associated with marine invertebrates. These photosynthetic dinoflagellates drive primary production and play a central role in reef formation, yet they are highly sensitive to environmental stress [12]. The composition of coral symbiont communities is dynamic and influenced by environmental variability, host identity, and ecological history, highlighting the importance of symbiont specificity and environmental memory in determining coral resilience [13,14]. Advances in molecular and phylogenetic analyses have refined the taxonomy of Symbiodiniaceae, now comprising multiple genera, including *Symbiodinium*, *Breviolum*, *Cladocopium*, and *Durusdinium* [12,15,16,17]. Among these, *Cladocopium* and *Durusdinium* are commonly associated with corals, particularly in nearshore environments [18,19,20].

Reactive oxygen species (ROS) play a pivotal role in coral bleaching by mediating oxidative stress within the coral-algal symbiotic system [20,21,22]. ROS are primarily generated in the chloroplasts of symbiotic algae and can overwhelm antioxidant defense systems under stress conditions [21]. Excessive ROS accumulation disrupts photosynthesis, impairs metabolic pathways, and damages cellular structures, ultimately leading to the expulsion of symbionts through mechanisms such as apoptosis, exocytosis, and autophagy [21,22,23]. Nevertheless, coral bleaching is a multifactorial process involving complex cellular and physiological responses beyond ROS alone [22,24]. *Cladocopium* is the most widespread and dominant genus of symbiotic dinoflagellates, exhibiting high genetic and functional diversity, although many species are sensitive to thermal stress [2,15,25,26,27,28,29]. In contrast, species within the genus *Durusdinium* are generally more thermally tolerant and confer enhanced heat resistance to their coral hosts [23,30].

Nano-selenium (SeNPs), elemental selenium particles ranging from 1 to 100 nm, possess high surface area, enhanced bioavailability, and strong redox-modulating properties, which underpin their expanding applications in biomedicine, environmental remediation, and marine technologies [31,32]. Compared with conventional selenium forms, SeNPs exhibit 5–10 times higher antioxidant activity, effectively scavenging reactive oxygen species (ROS), such as hydroxyl radicals and superoxide anions, while simultaneously enhancing antioxidant enzyme systems (e.g., SOD and CAT) to maintain cellular redox homeostasis [33]. However, these effects are strongly concentration-dependent; while low doses promote metabolic activity and stress resilience, excessive SeNP exposure can disrupt redox balance, induce oxidative stress, and impair cellular morphology, photosynthesis, and overall physiological performance in microalgae [34]. Microalgae themselves are efficient platforms for SeNP biosynthesis, as demonstrated in *Chlorella* and *Spirulina*, which can transform inorganic selenium into organic and protein-bound forms, further enhancing its biological functionality [35]. In aquaculture systems, dietary supplementation with SeNPs has been shown to improve antioxidant capacity, immune response, and overall physiological health in fish species [36]. Moreover, green synthesis approaches, such as plant-extract-mediated production, significantly improve the biocompatibility, environmental safety, and functional performance of SeNPs by replacing toxic chemical reagents with sustainable biological resources [37,38].

Despite these advances, the role of nano-selenium in regulating the physiology of coral symbiotic dinoflagellates remains poorly understood [39,40]. In particular, species-specific responses, redox regulation mechanisms, and links to biofilm-relevant processes have not been systematically investigated. Given that biofouling is initiated by microalgal colonization governed by redox-dependent metabolic activity, understanding how SeNPs influence oxidative balance and physiological responses in these organisms is critical for both coral conservation and the development of environmentally friendly antifouling strategies.

Therefore, this study employed a green synthesis approach using banana peel extracts to produce nano-selenium, followed by comprehensive characterization and evaluation of its effects on the growth, photosynthetic performance, antioxidant systems, and osmotic regulation of *Cladocopium* sp. and *Durusdinium* sp. Specifically, this work aims to (i) characterize SeNP physicochemical properties, (ii) quantify concentration-dependent physiological and redox responses, and (iii) elucidate species-specific adaptive strategies, including growth-oriented versus tolerance-oriented responses under nano-Se exposure. Particular emphasis is placed on differences in selenium assimilation capacity, antioxidant system activation, and metabolic flux redistribution, which collectively determine redox homeostasis and cellular resilience. By linking these responses to redox-driven microalgal activity, this study further evaluates the potential of nano-selenium as a regulator of early-stage biofilm formation. Ultimately, the findings are expected to identify optimal nano-Se application thresholds and provide a mechanistic foundation for the targeted management of symbiotic dinoflagellates, contributing to sustainable coral aquaculture, improved marine infrastructure protection, and the development of eco-friendly antifouling technologies.

## 2. Results and Discussions

### 2.1. Green-Synthesized SeNPs Exhibit High Stability, Uniform Morphology, and Biomolecule-Mediated Surface Functionalization

**Figure 1:**
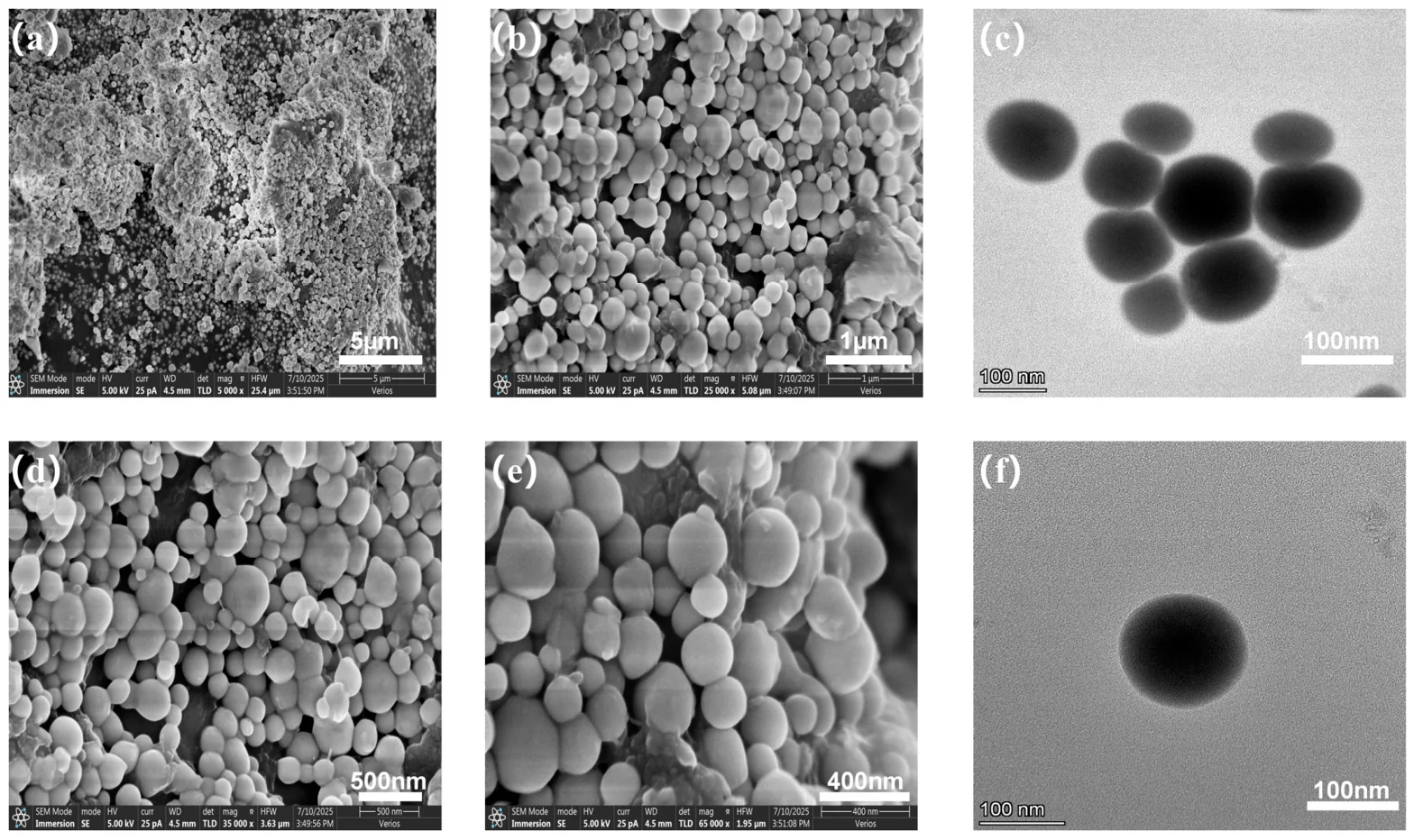
SEM (**a**,**b**,**d**,**e**) images of SeNPs at different magnifications, showing surface morphology and particle distribution; (**c**,**f**) TEM images showing primary particle size and internal structure. Scale bars are indicated in each image.

Scanning electron microscopy (SEM) is widely used to characterize the microstructure and surface morphology of nanomaterials [41]. As shown in Figure 1, the SEM images of nano-selenium synthesized using banana peel extract reveal that the particles are well dispersed, with no evident large-scale aggregation. At higher magnification, the nanoparticles appear as predominantly spheroids with smooth surfaces and no visible impurities [42], suggesting that biomolecules from the banana peel extract form a uniform coating on the nanoparticle surface.

Transmission electron microscopy (TEM), which provides higher resolution imaging, further confirms these observations [43]. The TEM images show that the synthesized SeNPs are predominantly spherical, with well-defined boundaries, smooth edges, and a relatively narrow size distribution, indicating good monodispersity and minimal aggregation.

**Figure 2:**
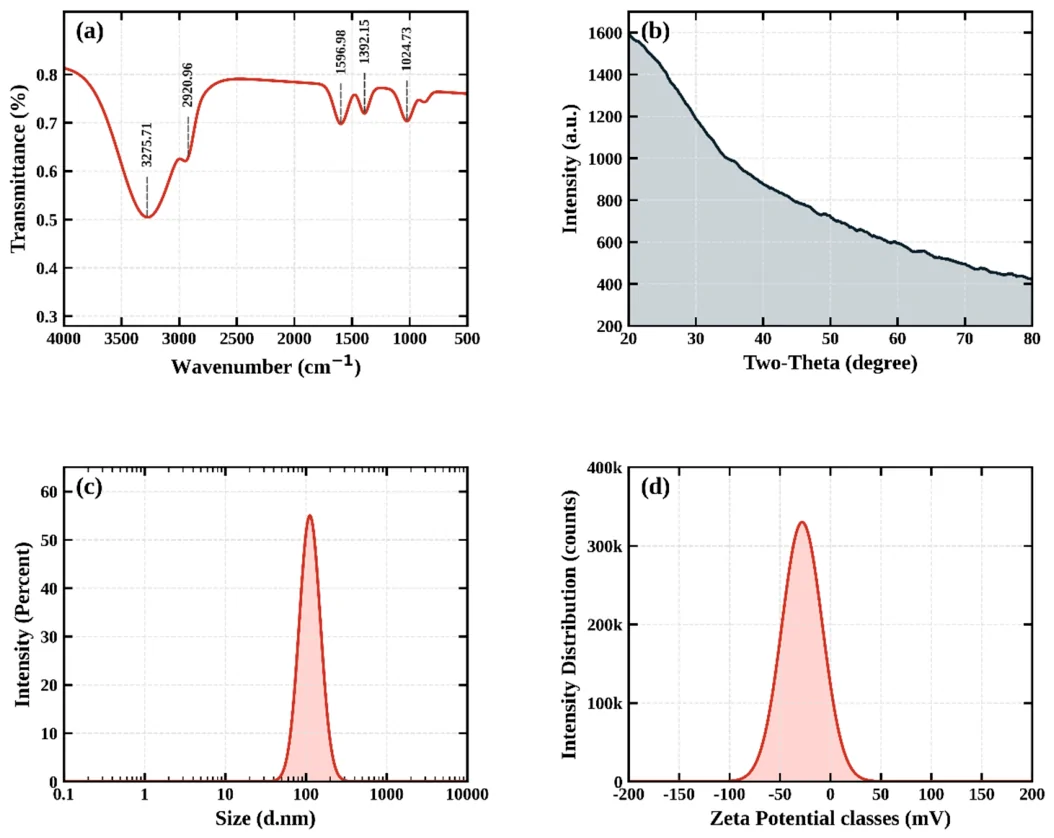
FTIR spectra (**a**), XRD patterns (**b**), particle size distribution (**c**), and zeta potential (**d**) of SeNPs.

Fourier transform infrared spectroscopy (FTIR) was employed to identify functional groups and chemical bonds associated with the synthesized SeNPs [44,45]. As shown in Figure 2a, a broad peak at 3275.71 cm^−1^ corresponds to O–H stretching vibrations, indicating the presence of hydroxyl-containing biomolecules on the nanoparticle surface. Peaks at 2920.96 cm^−1^ C–H asymmetric stretching and 1596.98 cm^−1^ are attributed to C=O stretching vibrations of amide groups, suggesting protein binding. The peak at 1392.15 cm^−1^ represents C–O stretching vibrations, associated with hydroxyl or carbonyl groups, while the peak at 1024.73 cm^−1^ corresponds to C–O bending vibrations, further confirming the presence of polysaccharides and other biomolecules. Together, these FTIR features indicate that proteins, polysaccharides, and other plant-derived biomolecules are involved in both the reduction of selenium precursors and the stabilization (capping) of SeNPs, contributing to their colloidal stability.

The X-ray diffraction (XRD) pattern (Figure 2b) shows a broad and diffuse peak, indicating the amorphous nature of the synthesized nano-selenium 51. The diffuse scattering with decreasing intensity across the 20° range is characteristic of amorphous materials, suggesting low crystallinity of the SeNPs.

Dynamic light scattering (DLS) analysis (Figure 2c) reveals a narrow, unimodal particle size distribution, indicating good monodispersity. The hydrodynamic diameter was centered at approximately 112.9 nm (range ~100 to 200 nm). This size range is consistent with TEM observations, although slightly larger due to hydration layers measured in solution. The narrow distribution further confirms the absence of significant aggregation. TEM imaging was used to assess the morphology and dry-state primary particles, whereas DLS measured the hydrodynamic diameter of the particles in aqueous suspension. The DLS value therefore reflects the particle core together with the surface-associated biomolecular coating, hydration layer, and any particle association present in suspension.

The zeta potential distribution (Figure 2d) shows an average value of −44.6 mV, with a narrow and unimodal profile, indicating high colloidal stability. According to the commonly accepted stability threshold (±30 mV), the strong negative surface charge provides sufficient electrostatic repulsion between particles, preventing aggregation and ensuring stable dispersion.

Overall, the combined SEM, TEM, FTIR, XRD, and DLS analyses demonstrate that the green-synthesized SeNPs possess stable morphology, favorable dispersion, and biomolecule-mediated surface functionalization, making them suitable for biological interaction studies.

### 2.2. Nano-Selenium Induces a Hormetic Growth Response with Species-Specific Proliferation Strategies

From an applied perspective, nano-selenium (nano-Se) regulates algal growth and redox balance in a concentration-dependent and species-specific manner in both *Cladocopium* and *Durusdinium*, with important implications for marine biofouling control.

The biphasic response observed in this study indicates that nano-Se can promote physiological stability at low concentrations while suppressing excessive growth at higher levels. Unlike conventional biocidal antifouling agents, nano-Se regulates cellular processes through redox-mediated modulation rather than direct toxicity, offering a potentially more sustainable strategy for incorporation into antifouling coatings and nano-enabled marine protection systems [46,47].

**Figure 3:**
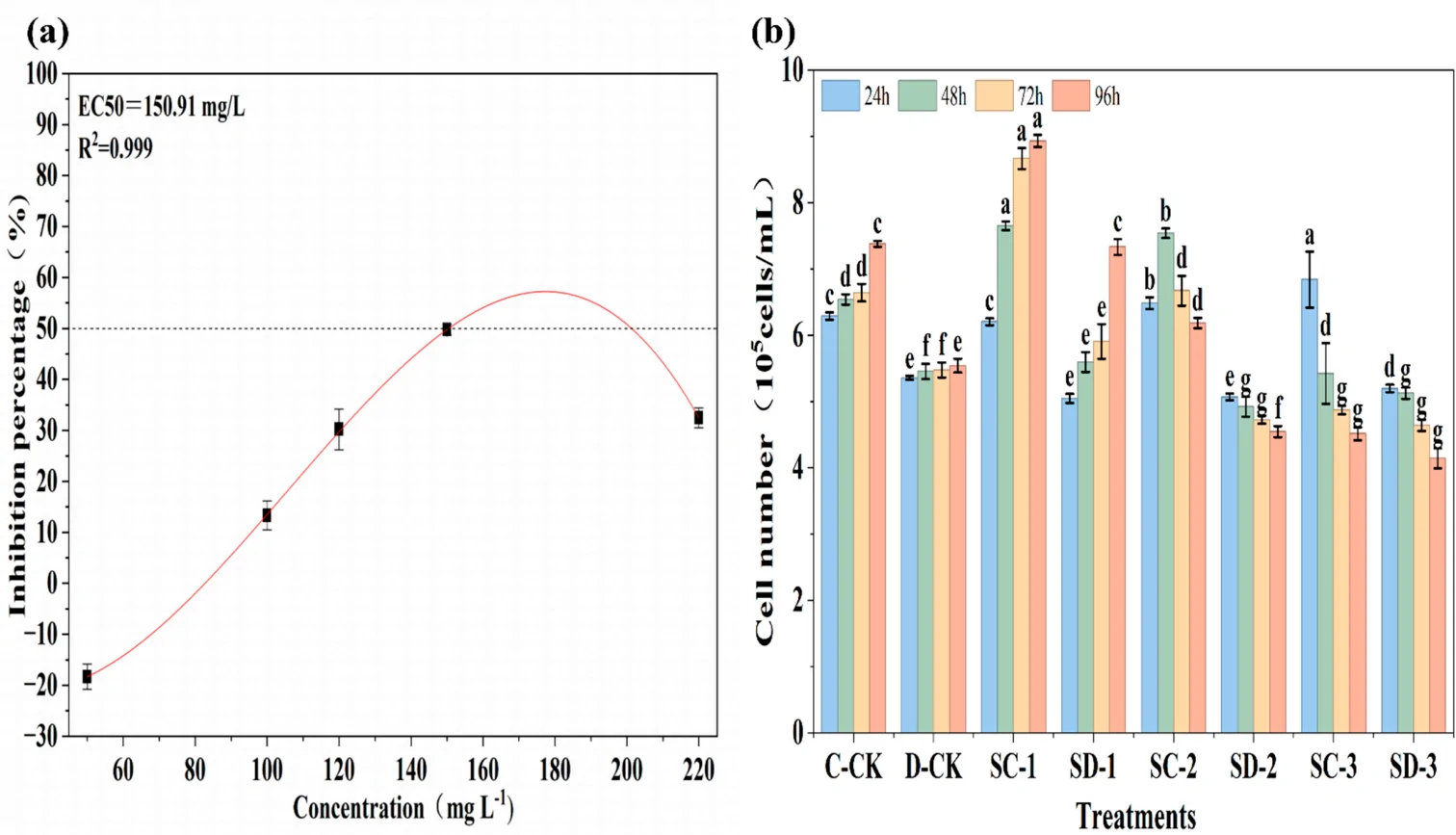
Dose–response curve (**a**) and changes in cell density (**b**) of *Cladocopium* sp. and *Durusdinium* sp. treated with Se-NPs over 96 h. Different letters indicate statistically significant differences (*p* < 0.05). The bar chart represents the mean ± SD (*n* = 3). C-CK and D-CK refer to the blank control groups of *Cladocopium* sp. and *Durusdinium* sp., respectively; SC-1 to SC-3 represent the three SeNP concentrations applied to *Cladocopium* sp.; and SD-1 to SD-3 represent the three SeNP concentrations applied to *Durusdinium* sp.

As shown in Figure 3a, the 96 h concentration-response analysis for *Cladocopium* sp. yielded a nominal EC_50_ of 150.91 mg L^−1^. However, the decline in inhibition at 220 mg L^−1^ indicates a deviation from a strictly monotonic dose–response pattern at the highest tested concentration. The initial inoculation density for both species was 5 × 10^5^ cells mL^−1^. As shown in Figure 3b, the low-dose treatment (SC-1) significantly enhanced *Cladocopium* growth, reaching peak biomass at 96 h, which was 47.5% higher than the control. In contrast, the high-dose treatment (SC-3) markedly inhibited growth, resulting in a 21.3% reduction in biomass at 96 h. Compared with *Cladocopium*, *Durusdinium* exhibited a weaker response to nano-selenium. The SD-1 treatment induced only a slight growth promotion, whereas SD-2 and SD-3 treatments caused continuous growth inhibition throughout the experimental period, with biomass reaching its lowest level at 96 h. These results suggest that *Durusdinium* adopts a conservative survival strategy, prioritizing stress tolerance over rapid proliferation.

These findings align with the hormesis framework [48,49], where low-dose exposure enhances cellular activity while high-dose exposure leads to toxicity. The contrasting patterns indicate that *Cladocopium* sp. prioritizes growth, whereas *Durusdinium* sp. emphasizes physiological stability under stress. This divergence in growth response suggests that nano-Se exposure reveals intrinsic differences in resource allocation strategies between the two symbionts, with implications for their ecological roles and contributions to early-stage biofilm formation.

### 2.3. Photosynthetic Performance Is Enhanced at Low Nano-Se but Impaired at High Concentrations, with Greater Resilience in Durusdinium

**Figure 4:**
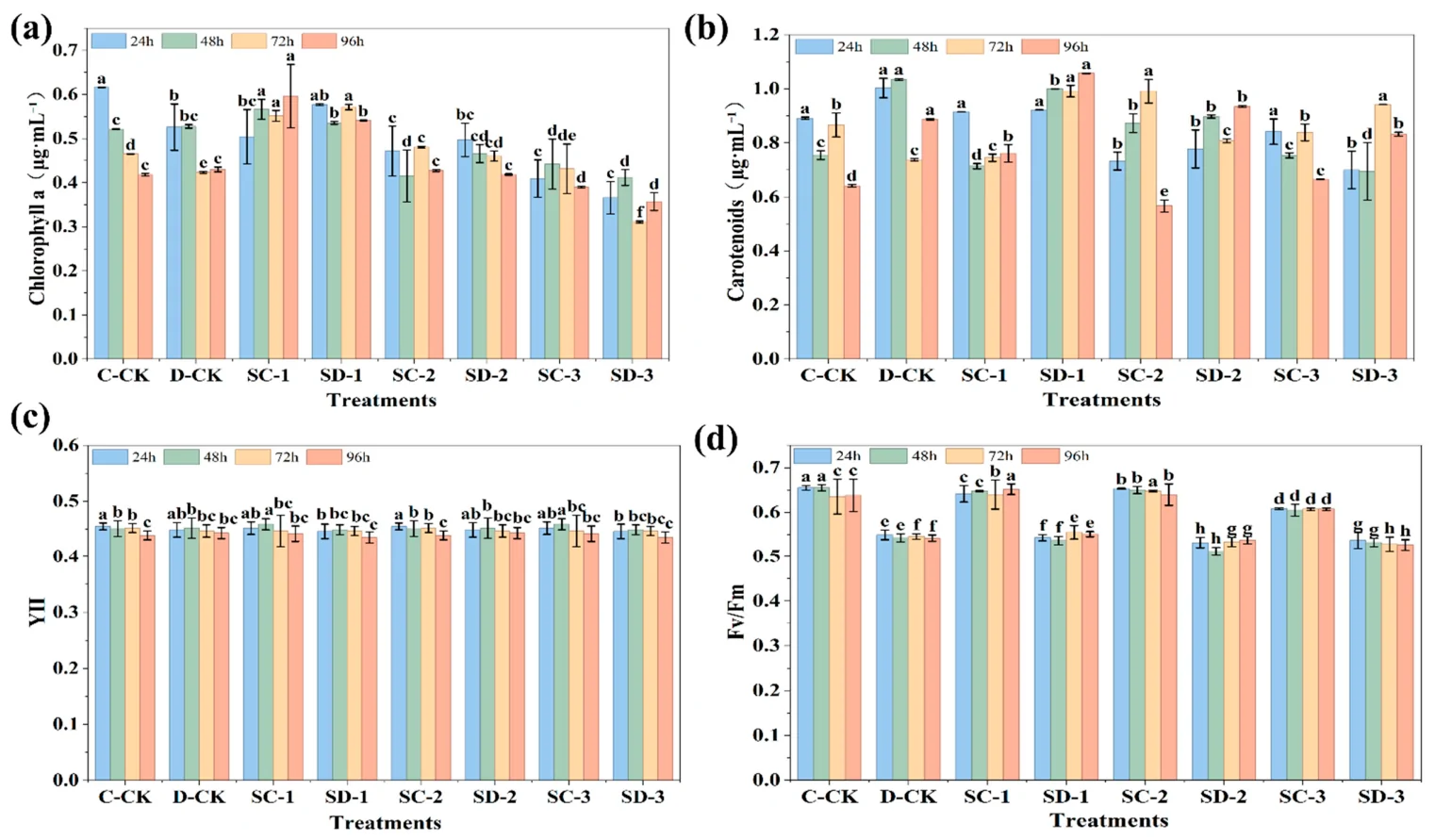
Effects of Se-NPs on *Cladocopium* sp. and *Durusdinium* sp. chlorophylls (**a**), carotenoids (**b**), YII (**c**), and Fv/Fm (**d**). Different letters indicate statistically significant differences (*p* < 0.05). The bar chart represents the mean ± SD (*n* = 3). C-CK and D-CK refer to the blank control groups of *Cladocopium* sp. and *Durusdinium* sp., respectively; SC-1 to SC-3 represent the three SeNP concentrations applied to *Cladocopium* sp.; and SD-1 to SD-3 represent the three SeNP concentrations applied to *Durusdinium* sp.

Nano-selenium exerted clear concentration-dependent effects on photosynthetic performance in both species. *Cladocopium* exhibited a typical hormetic response, with stimulation at low concentrations and inhibition at high concentrations. Figure 4: In control conditions, chlorophyll a content declined from 0.616 ± 0.0005 to 0.418 ± 0.0028 mg cell^−1^ over 96 h, accompanied by a gradual decrease in carotenoids. Under low-dose treatment (SC-1), photosynthetic pigment synthesis was significantly enhanced. Chlorophyll a remained stable between 0.54 and 0.60 mg cell^−1^ from 48 to 96 h, reaching 0.596 ± 0.061 mg cell^−1^ at 96 h. Carotenoid content increased to 0.76 ± 0.03 mg cell^−1^. Additionally, Y(II) and Fv/Fm values were significantly higher than the control, indicating enhanced PSII efficiency and improved photosynthetic performance.

In contrast, higher concentrations (SC-2 and SC-3) strongly inhibited the photosynthetic system of *Cladocopium*. Chlorophyll a decreased to 0.39 ± 0.005 mg cell^−1^ at later stages, significantly lower than the control. Carotenoid synthesis became unstable, with a transient peak at 72 h in SC-2 followed by a decline to 0.56 ± 0.02 mg cell^−1^ at 96 h. In SC-3, carotenoids remained consistently low (0.67 ± 0.001 mg cell^−1^). Both Y(II) and Fv/Fm declined markedly, indicating functional damage to the PSII reaction center, consistent with increased oxidative stress and pigment degradation.

*Durusdinium* exhibited a more stable and conservative response. In the control group, chlorophyll a (0.42–0.55 μg mL^−1^) and carotenoids (0.73–1.04 μg mL^−1^) remained relatively constant. Under SD-1 treatment, chlorophyll a increased to 0.54–0.58 μg mL^−1^, and carotenoids peaked at 1.01 ± 0.03 μg mL^−1^ at 96 h. However, Y(II) and Fv/Fm showed no significant enhancement, remaining comparable to the control. At higher concentrations (SD-2 and SD-3), *Durusdinium* exhibited only moderate inhibition. Chlorophyll a decreased to 0.31–0.39 μg mL^−1^, and carotenoids remained within 0.74–0.94 μg mL^−1^. Importantly, Y(II) and Fv/Fm remained relatively stable, indicating that PSII function was not severely impaired. This suggests that *Durusdinium* possesses greater tolerance to nano-selenium-induced stress compared with *Cladocopium*. These photosynthetic responses further support the divergent strategies between the two species [50,51]. At low concentrations, *Cladocopium* shows coordinated increases in chlorophyll a and carotenoids, indicating enhanced photosynthetic efficiency and protection of the photosynthetic apparatus [30]. However, at high concentrations, pigment synthesis becomes disrupted, and carotenoid-mediated photoprotection is insufficient, resulting in damage to the PSII complex and reduced growth. In contrast, *Durusdinium* maintains relatively stable chlorophyll a level and gradually accumulates carotenoids, suggesting a more robust and conservative photosynthetic regulation strategy.

### 2.4. Contrasting Antioxidant Coordination Determines Differential Oxidative Stress Tolerance Between Species

**Figure 5:**
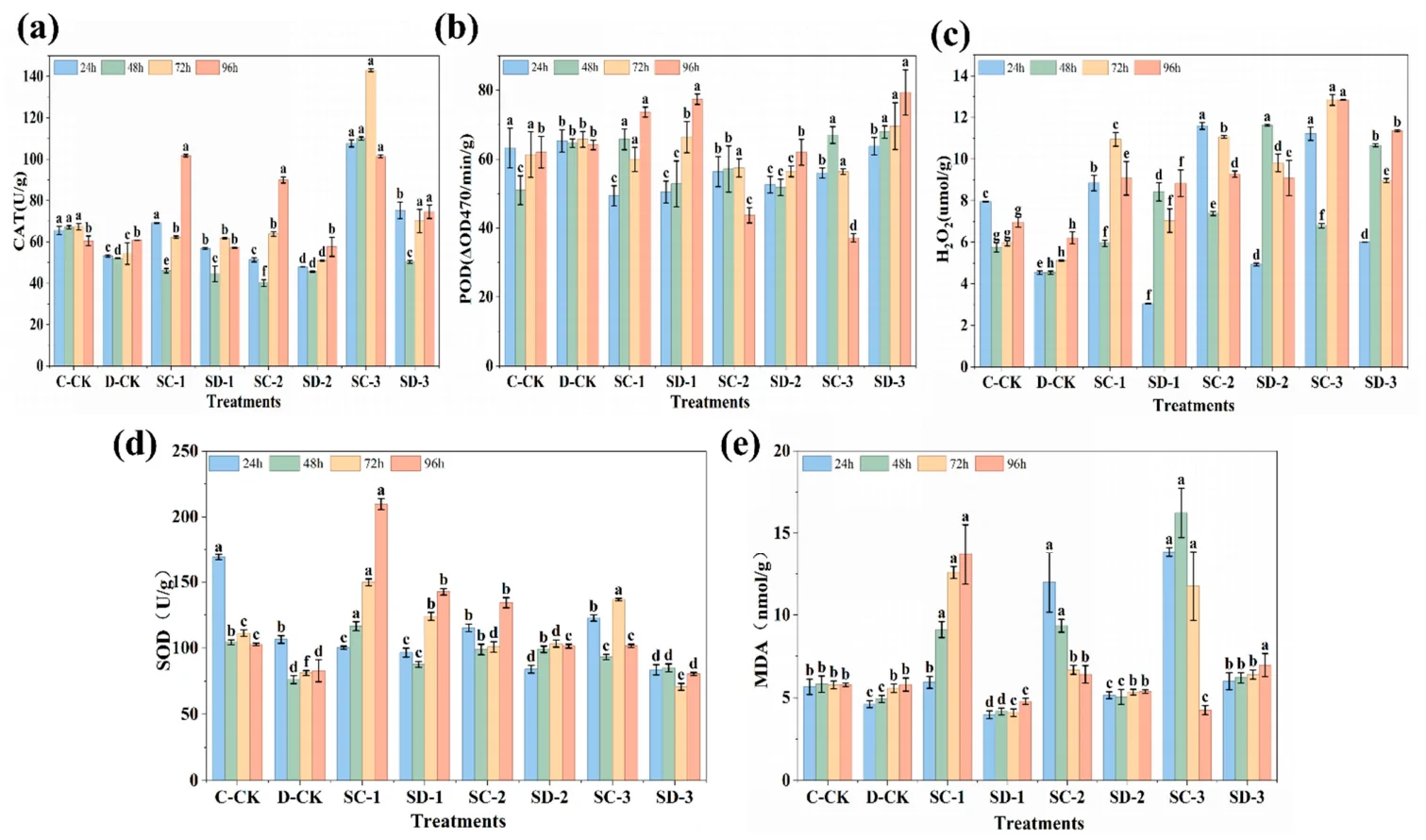
Effects of SeNPs on antioxidant enzyme activities CAT (**a**), POD (**b**), H_2_O_2_ (**c**), SOD (**d**), and MDA (**e**). Different letters indicate statistically significant differences (*p* < 0.05). The bar chart represents the mean ± SD (*n* = 3). C-CK and D-CK refer to the blank control groups of *Cladocopium* sp. and *Durusdinium* sp., respectively; SC-1 to SC-3 represent the three SeNP concentrations applied to *Cladocopium* sp.; and SD-1 to SD-3 represent the three SeNP concentrations applied to *Durusdinium* sp.

Catalase (CAT) activity in *Cladocopium* showed a clear time- and concentration-dependent increase (Figure 5a). Although no significant difference was observed at 24 h, CAT activity increased with prolonged exposure. At 96 h, CAT activity in the SC-1, SC-2, and SC-3 treatments increased by 33.7%, 50.4%, and 69.5%, respectively, compared with the control. In contrast, CAT activity in *Durusdinium* remained relatively stable, with only the high-dose (SD-3) treatment showing a moderate increase (44.7%) at 72 h.

Peroxidase (POD) activity increased significantly in *Cladocopium* under all nano-selenium treatments, particularly at 72 h, with increases of 17.3%, 14.5%, and 20.4% for SC-1, SC-2, and SC-3, respectively (Figure 5b). In *Durusdinium*, only SD-1 and SD-3 treatments showed moderate increases (8.4% and 17.6%), while SD-2 remained comparable to the control. Overall, POD activity increased with exposure time in both species, although responses were variable in *Cladocopium*.

Hydrogen peroxide (H_2_O_2_) accumulation exhibited pronounced interspecific differences (Figure 5c). In *Cladocopium*, low-dose treatment maintained relatively stable or slightly reduced H_2_O_2_ levels, whereas moderate and high concentrations led to continuous accumulation, reaching 12.84 μmol g^−1^ at 96 h (1.9-fold higher than the control), indicating severe oxidative stress. In contrast, *Durusdinium* maintained lower basal H_2_O_2_ levels, with only transient increases under higher concentrations, suggesting more efficient ROS scavenging capacity.

Superoxide dismutase (SOD) activity also varied between species (Figure 5d). In *Cladocopium*, SOD activity increased significantly under SC-1 and SC-3 treatments, while SC-2 showed a consistent upward tend at 72 h. At 96 h, SC-1 and SC-2 increased by 52.3% and 30.6%, respectively. In *Durusdinium*, SOD activity was markedly enhanced under SD-1 and SD-2, increasing by 22.1% and 5.4%, respectively, whereas SD-3 showed a downward trend.

Malondialdehyde (MDA) content, an indicator of lipid peroxidation, showed strong concentration-dependent responses (Figure 5e). In *Cladocopium*, low-dose treatment (SC-1) induced a gradual elevation of MDA levels, while higher concentrations (SC-2 and SC-3) significantly increased MDA accumulation, with SC-3 peaking at 48 h before amarked decline at 96 h, indicating severe membrane damage. In contrast, *Durusdinium* maintained lower MDA levels across treatments, with reductions of 30.4%, 5.7%, and 23.0% in SD-1, SD-2, and SD-3, respectively, suggesting effective mitigation of oxidative damage. Overall, antioxidant enzyme activities (SOD, POD, and CAT) were inversely associated with MDA accumulation in both species, indicating that coordinated enzymatic responses effectively mitigate lipid peroxidation and oxidative damage [52,53,54].

In *Cladocopium*, high nano-selenium concentrations induced strong CAT activation, while SOD and POD exhibited fluctuating responses. This imbalance in antioxidant enzyme coordination limited effective ROS detoxification, leading to increased MDA accumulation and membrane damage, and growth inhibition. Conversely, *Durusdinium* maintained a more balanced antioxidant response. SOD activity was strongly induced at low concentrations and moderated at higher concentrations to maintain metabolic efficiency, while CAT and POD activities increased steadily. This coordinated response effectively limited lipid peroxidation and maintained intracellular redox homeostasis, reflecting a tolerance-based survival strategy [55].

These results suggest that antioxidant coordination, rather than the magnitude of individual enzyme activities, is the key determinant of oxidative stress tolerance under nano-Se exposure. This coordination enables *Durusdinium* to maintain redox balance more effectively than *Cladocopium*, thereby supporting its greater resilience under elevated nano-selenium stress.

### 2.5. Distinct Osmolyte Accumulation Patterns Reflect Rapid-Response Versus Baseline-Stability Strategies

**Figure 6:**
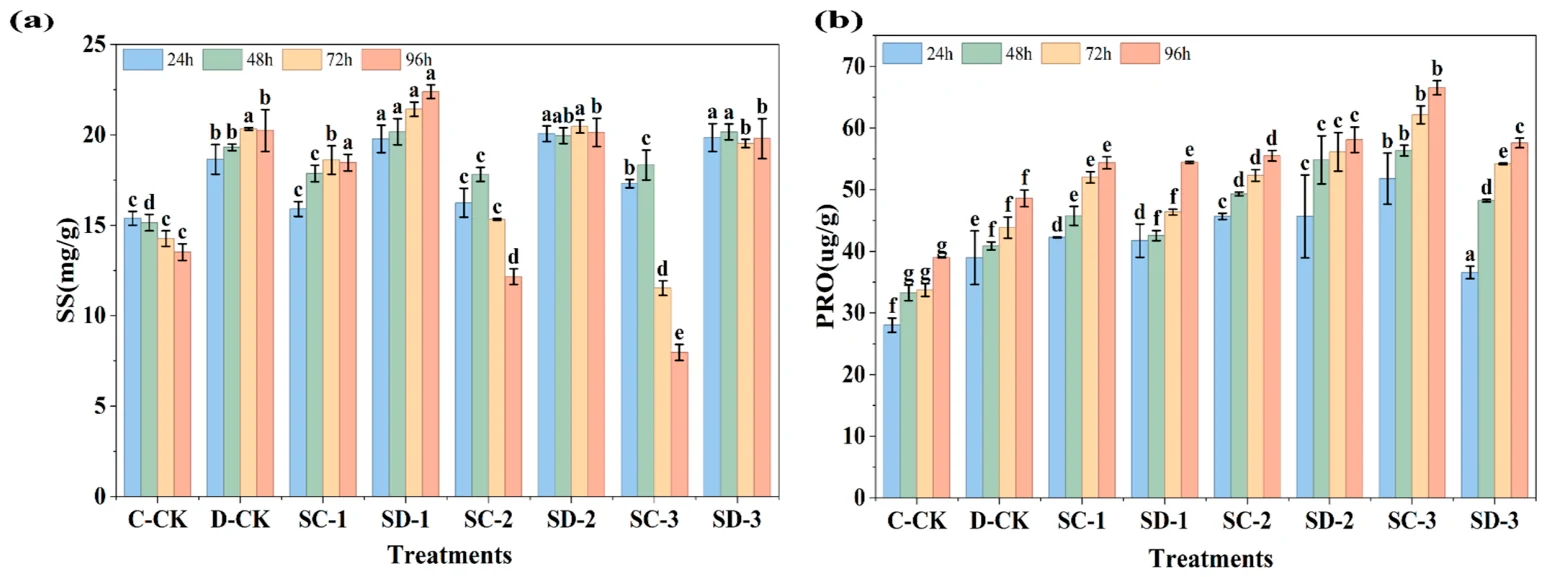
Effects of Se-NPs on *Cladocopium* sp. and *Durusdinium* sp. SS (**a**) and PRO (**b**). Different letters indicate statistically significant differences (*p* < 0.05). The bar chart represents the mean ± SD (*n* = 3). C-CK and D-CK refer to the blank control groups of *Cladocopium* sp. and *Durusdinium* sp., respectively; SC-1 to SC-3 represent the three SeNP concentrations applied to *Cladocopium* sp.; and SD-1 to SD-3 represent the three SeNP concentrations applied to *Durusdinium* sp.

As shown in Figure 6a, low-concentration nano-selenium markedly stimulated soluble sugar (SS) accumulation in both species. At 72 h, SS content in *Cladocopium* (SC-1) reached 18.61 mg g^−1^, representing a 30.4% increase compared with the control, whereas *Durusdinium* reached 21.42 mg g^−1^, corresponding to a 5.4% increase. At 96 h, SS accumulation further increased, with *Cladocopium* reaching 18.47 mg g^−1^ (36.6% higher than the control) and *Durusdinium* reaching 22.39 mg g^−1^ (10.6% higher than the control). Although *Durusdinium* maintained a higher absolute SS content than *Cladocopium*, the relative increase was consistently greater in *Cladocopium*, with differences reaching up to 26% at 96 h. These results indicate that *Cladocopium* exhibits a more sensitive and rapid osmotic response to nano-selenium stress through enhanced SS accumulation, whereas *Durusdinium* maintains a more stable osmotic balance with higher basal SS levels and gradual increases over time.

Proline accumulation showed similar but species-specific patterns (Figure 6b). At 72 h, PRO content in *Cladocopium* under low-dose treatment reached 52.03 mg g^−1^, representing a 54.2% increase compared with the control. In contrast, *Durusdinium* exhibited a modest increase to 46.40 mg g^−1^ (5.8% higher than the control), although its absolute PRO content remained relatively high. At 96 h, PRO accumulation continued in both species. *Cladocopium* reached 54.37 mg g^−1^ (39.4% higher than the control), maintaining elevated levels despite a slight decline compared to 72 h. *Durusdinium* increased to 54.46 mg g^−1^, corresponding to a 12.0% increase over the control and a 6.2% increase compared to 72 h, slightly exceeding *Cladocopium* in absolute terms.

These osmoregulatory responses reinforce the contrasting strategies between the two species [56]. Low concentrations of nano-selenium promoted the accumulation of proline and soluble sugars in both species, with *Cladocopium* exhibiting a rapid and high-amplitude increase, indicative of a sensitive and inducible stress-response system. In contrast, *Durusdinium* maintained higher baseline levels of osmolytes with gradual increases, supporting stable osmotic balance with lower metabolic investment. Together with antioxidant responses, this osmotic regulation contributes to differential stress tolerance between the two species.

These findings suggest that osmolyte accumulation functions as a complementary mechanism to antioxidant defenses, enabling cells to maintain intracellular homeostasis under nano-Se-induced redox perturbation. In particular, the rapid inducible response in *Cladocopium* contrasts with the baseline-stability strategy of *Durusdinium*, further highlighting their distinct resource allocation strategies under stress.

### 2.6. Redox-Mediated Regulation by SeNPs Underpins Species-Specific Survival Strategies and Antifouling Potential

**Figure 7:**
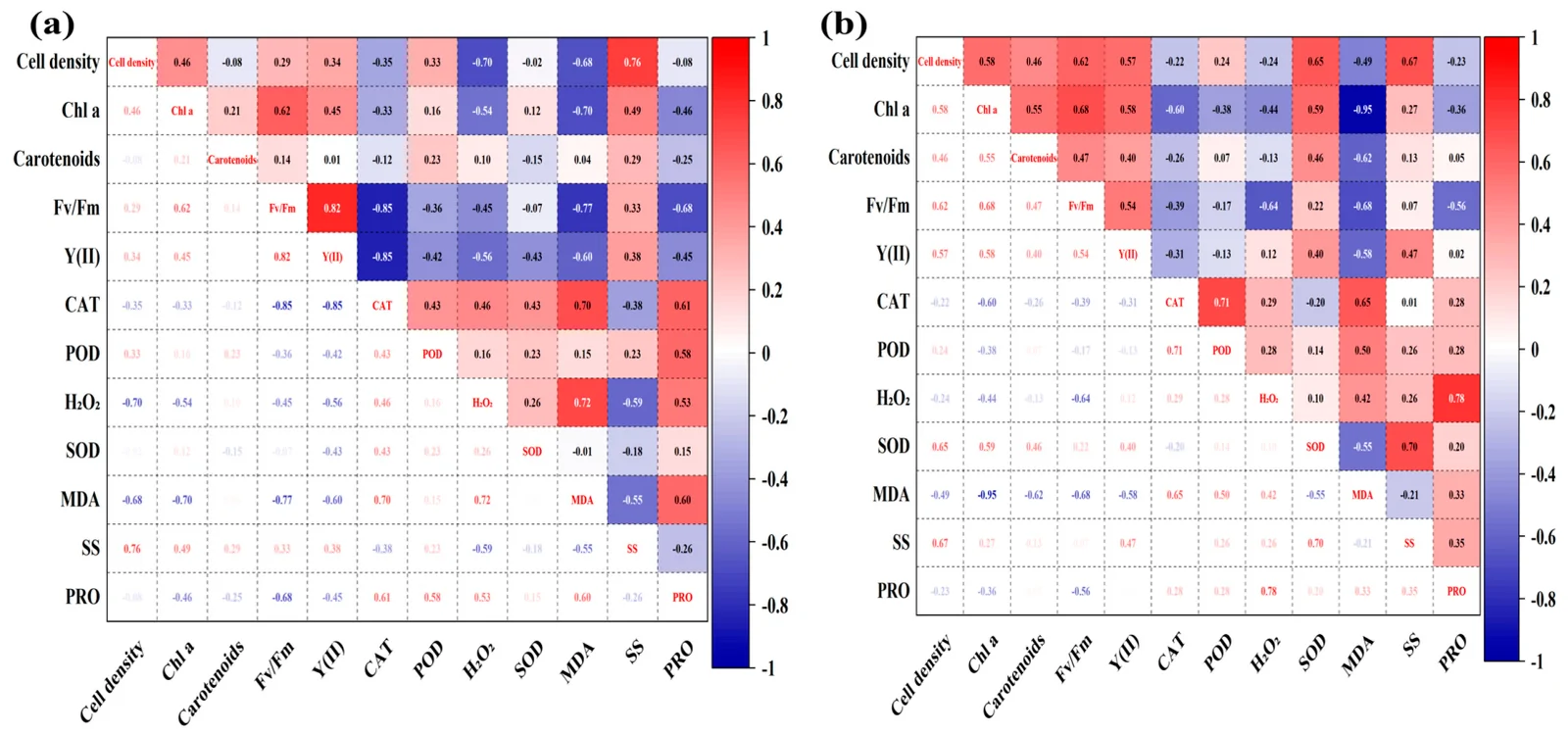
Comparison of the correlation heat maps of physiological indicators of *Cladocopium* sp. (**a**) and *Durusdinium* sp. (**b**) under Se-NPs treatment. Red and blue, respectively, represent positive correlation and negative correlation.

To illustrate the different survival strategies of *Cladocopium* and *Durusdinium* under nano-selenium treatment, this paper conducted a Pearson correlation analysis on 12 physiological indicators (cell density, chlorophyll a, carotenoids, Fv/Fm, Y(II), CAT, POD, H_2_O_2_, SOD, MDA, soluble sugar, and proline), and presented the correlation coefficient matrix of the two species in the form of a heatmap (Figure 7a for *Cladocopium*, Figure 7b for *Durusdinium*).

In *Cladocopium*, cell density and Fv/Fm (r = 0.62) and Y(II) (r = 0.57) showed strong positive correlations and were moderately positively correlated with chlorophyll a (r = 0.58). Chlorophyll a was strongly negatively correlated with MDA (r = −0.95), indicating that membrane lipid peroxidation is closely associated with the degradation of photosynthetic pigments. H_2_O_2_ was strongly positively correlated with proline (r = 0.78) and soluble sugar (r = 0.70), indicating that ROS accumulation strongly induces osmotic regulation. CAT was strongly positively correlated with MDA (r = 0.65) and POD (r = 0.71), indicating that activation of antioxidant enzymes alone was insufficient to fully prevent membrane damage under high nano-selenium concentrations.

In contrast, the correlation between cell density and photosynthetic parameters in *Durusdinium* was much weaker (Fv/Fm: r = 0.14; Y(II): r = 0.34), indicating that growth and photosynthetic efficiency were largely decoupled. Cell density was moderately positively correlated with chlorophyll a (r = 0.46) and positively correlated with MDA (r = 0.33), whereas in *Cladocopium* it was negatively correlated (r = −0.49). This suggests that mild oxidative stress does not immediately inhibit growth but is accommodated through compensatory tolerance mechanisms.

The correlation between chlorophyll a and MDA in *Durusdinium* (r = −0.62) was also weaker than that in *Cladocopium*, suggesting that its photosynthetic pigments were better protected from lipid oxidative damage. The positive correlation between CAT and MDA in *Durusdinium* (r = 0.82) was stronger than that in *Cladocopium*, while overall MDA levels remained lower, indicating a more efficient and balanced antioxidant system capable of effectively clearing ROS and limiting membrane damage.

Additionally, the correlation between H_2_O_2_ and proline (r = 0.46) and soluble sugar (r = 0.42) in *Durusdinium* was weaker than that in *Cladocopium*, consistent with its higher basal osmolyte levels and reduced reliance on strong inducible responses under stress.

Overall, these correlation patterns clearly distinguish the stress response modes of the two dinoflagellates. *Cladocopium* adopted a photosynthetic-driven, growth-prioritizing strategy with a strong positive correlation between growth and photosynthetic parameters but exhibited high sensitivity to oxidative stress. *Durusdinium* adopted a tolerance-oriented strategy, in which growth is less tightly coupled to photosynthetic efficiency.

The concentration-dependent responses observed in the present study are broadly consistent with previous reports on the effects of selenium compounds in microalgae. Babazadeh et al. found that SeO_2_ produced a biphasic response in *Tetradesmus obliquus*, with lower concentrations (25–50 mg L^−1^) promoting growth and maintaining photosynthetic pigments, whereas higher concentrations (75–100 mg L^−1^) increased oxidative stress and inhibited growth [57]. Comparable hormetic patterns have also been reported for other selenium forms, in which moderate exposure supports photosynthetic activity and antioxidant defense, while excessive selenium disrupts redox homeostasis, promotes oxidative damage, and suppresses algal growth [58,59,60,61]. Our findings extend this general pattern to coral symbiotic dinoflagellates exposed to the purified SeNP preparation. *Cladocopium* sp. exhibited stronger growth and photosynthetic stimulation at the lower concentration but greater sensitivity at elevated exposure, whereas *Durusdinium* sp. maintained comparatively stable photosynthetic and oxidative-stress responses. Nevertheless, direct comparisons among studies should be made cautiously because selenium speciation, dissolution behavior, exposure duration, culture conditions, and species-specific uptake can substantially influence algal responses. Therefore, the present results indicate a broadly similar concentration-dependent response pattern but do not demonstrate that SeNPs and dissolved selenium compounds act through identical mechanisms. The nominal SeNP preparation concentrations used in this study (50–150 mg L^−1^) were intentionally selected to define the short-term concentration-response window and identify the transition from physiological stimulation to growth inhibition under controlled laboratory conditions. Accordingly, the lower tested concentration should be interpreted as the most favorable treatment within the experimental range rather than as an environmentally representative or recommended application dose. These findings provide a useful toxicological baseline for subsequent studies conducted at lower, environmentally relevant concentrations and under coral nursery conditions. Future work should further refine the exposure range by incorporating time-resolved measurements of particle stability, dissolution, selenium bioavailability, accumulation, host-symbiont performance, and responses of non-target organisms.

Differences among previous studies further emphasize that algal responses to selenium depend on selenium form, nanoparticle composition, surface chemistry, exposure duration, salinity, and species-specific physiological capacity. Singh et al., for example, reported that the halotolerant microalga *Dunaliella salina* tolerated 50 mg L^−1^ selenite under high-salinity conditions through enhanced glutathione metabolism, mitochondrial redox regulation, and increased ascorbate peroxidase and glutathione peroxidase activities [62]. Similarly, Lee et al. showed that the stress-tolerant microalga *Ettlia* sp. increased carotenoid production under adverse conditions, thereby supporting photosynthetic performance and limiting oxidative damage [63]. These findings are consistent with the comparatively stable antioxidant activity, carotenoid regulation, and redox homeostasis observed in *Durusdinium* sp. under SeNP exposure, whereas *Cladocopium* sp. showed stronger low-dose stimulation but greater sensitivity at the higher tested concentrations. Overall, the results indicate that selenium can exert beneficial regulatory effects within a species-specific concentration range, while the magnitude and direction of the response are shaped by exposure intensity, duration, and inherent physiological tolerance [64].

The purified SeNP preparation produced clear concentration-dependent changes in intracellular redox regulation in both symbiotic dinoflagellates. At the lower tested concentrations, exposure was associated with enhanced antioxidant enzyme activity, reduced lipid peroxidation, improved photosynthetic performance, and greater osmolyte accumulation, collectively supporting cellular homeostasis and growth. In contrast, higher concentrations disrupted redox balance, increased oxidative damage, impaired photosynthetic function, and inhibited cell proliferation.

Although the present study demonstrates clear concentration and species-dependent responses to the purified SeNP preparation, the absence of extract-only and sodium selenite controls limits the ability to attribute these responses exclusively to nanoparticulate selenium. Although the SeNP preparation was purified through three consecutive centrifugation and washing cycles with deionized water, and a medium-only control was included, residual dissolved selenium, unreacted selenite, and extract-derived biomolecules were not analytically quantified. Their possible contributions to the observed physiological responses therefore cannot be excluded. Accordingly, the present results should be interpreted as responses to the purified SeNP preparation rather than as effects attributable exclusively to nanoparticulate selenium. Future studies should include processed banana peel extract controls, equivalent-concentration Na_2_SeO_3_ controls, and direct measurements of residual selenium species and nanoparticle dissolution to establish SeNP-specific effects more conclusively.

Overall, the two dinoflagellates exhibited distinct concentration-dependent response patterns. *Cladocopium* sp. showed stronger growth and photosynthetic stimulation at lower SeNP concentrations but was more sensitive to high-dose exposure. In contrast, *Durusdinium* sp. displayed a more conservative response characterized by greater maintenance of photosynthetic function, antioxidant balance, and osmotic stability under elevated exposure. These contrasting patterns indicate that *Cladocopium* sp. follows a more growth-oriented strategy, whereas *Durusdinium* sp. relies more strongly on tolerance and homeostatic maintenance. Taken together, the findings suggest that the purified SeNP preparation acts as a redox-active modulator of cellular physiology rather than functioning solely as a direct ROS scavenger. Further studies should monitor particle size, zeta potential, aggregation, sedimentation, and dissolution throughout the exposure period under conditions matching the biological assays. Such measurements, together with the additional controls described above, will be necessary to determine the effective exposure dose and clarify the relative contributions of particulate selenium, dissolved selenium species, and plant-derived surface compounds.

## 3. Materials and Methods

### 3.1. Green Synthesis of Selenium Nanoparticles (SeNPs)

Fresh banana peels were collected locally (Haikou, China), thoroughly washed with deionized water, oven-dried at 60 °C, cooled, and ground into a fine powder. The powder was mixed with deionized water at a ratio of 1:10 (*w*/*v*) [65] and stirred at 60 °C and 200 rpm for 2 h using a constant-temperature magnetic stirrer. The mixture was then centrifuged at 7155× *g* for 15 min at 25 °C. Following three centrifugation and washing cycles, the recovered SeNP preparation was freeze-dried to obtain a dry powder. The powder was weighed gravimetrically and stored at 4 °C until use [66]. The recovered mass represented the purified biogenic SeNP preparation, including nanoparticle-associated capping biomolecules, rather than analytically verified elemental selenium.

For nanoparticle synthesis, the extract was continuously stirred at 50 °C, followed by the addition of 0.1 mol L^−1^ sodium selenite solution at a volume ratio of sodium selenite to extract of 1:2. The pH of the mixture was adjusted to 7.0 using 0.1 mol L^−1^ NaOH. The reaction mixture was incubated in the dark on a shaker for 12 h. A color change to red indicated the formation of SeNPs. The nanoparticles were subsequently purified by centrifugation (11,180× *g*, 15 min, 25 °C, three washing cycles with deionized water) and then re-dispersed in sterile deionized water [67]. All syntheses were performed in triplicate to ensure reproducibility.

### 3.2. Physicochemical Characterization of SeNPs

The morphology of the synthesized SeNPs was examined using scanning electron microscopy (SEM; Verios G4 UC, Thermo Fisher Scientific, Brno, Czech Republic and transmission electron microscopy (TEM; Spectra 300, Thermo Fisher Scientific, Eindhoven, The Netherlands). For SEM analysis, the dried SeNP samples were sputter-coated with gold before observation. For TEM analysis, the SeNP suspension was drop-cast onto carbon-coated copper grids and dried at room temperature prior to imaging.

Surface-associated functional groups were analyzed using Fourier-transform infrared spectroscopy (FTIR; Nicolet iS50, Thermo Fisher Scientific, Waltham, MA, USA) in attenuated total reflectance mode over a scan range of 4000–500 cm^−1^, at a resolution of 4 cm^−1^ with 32 scans. Crystallinity was assessed by X-ray diffraction (XRD; SmartLab, Rigaku, Akishima, Tokyo, Japan) over a 2θ range of 20–80°.

Hydrodynamic diameter, particle-size distribution, and polydispersity index were measured by dynamic light scattering, while surface charge was determined by zeta-potential analysis using a Zetasizer Nano ZSE (Malvern Panalytical, Malvern, UK). Freeze-dried SeNP powder was dispersed in deionized water using an ultrasonic disperser. Deionized water was used as the dispersant, and measurements were performed at 25 °C after a 2 min equilibration period. All DLS and zeta-potential measurements were conducted in triplicate.

### 3.3. Algal Culture Conditions

The dinoflagellate strains *Cladocopium* sp. and *Durusdinium* sp. were kindly provided by Prof. Senjie Lin from the Department of Marine Sciences, University of Connecticut, Groton, CT, USA. Cultures were maintained in L1 medium prepared with 0.22 μm-filtered and autoclaved seawater under a light intensity of 5000 lux (≈75 μmol photons m^−2^ s^−1^). Algal cells in the logarithmic growth phase were used for experiments under a 12 h light:12 h dark photoperiod.

Culture conditions were maintained at 26 ± 1 °C and a salinity of 35 PSU. Flasks were gently shaken 2–3 times daily to prevent cell aggregation and sedimentation. Prior to the experiment, all cultures were acclimated under these conditions to ensure physiological stabilization.

### 3.4. Cell Density Determination

Algal suspensions were diluted to generate a range of cell densities. For fixation, 100 μL of formaldehyde was added to 1 mL of algal suspension. Cell density was determined using a plankton counting chamber (22 × 26 × 0.5 mm) under a light microscope, with each sample counted in triplicate.

Optical density (OD_750_) was measured using a Multiskan SkyHigh microplate reader (Thermo Fisher Scientific, Waltham, MA, USA), and calibration curves were established to relate OD_750_ to cell number:

*Cladocopium* sp.:

$$Cell\;density\;\left(cells\;mL^{-1}\right)=1.116\times 10^{7}OD_{750}-5.11\times 10^{5}(R^{2}=0.998)$$

*Durusdinium* sp.:

$$Cell\;density\;\left(cells\;mL^{-1}\right)=3\times 10^{6}OD_{750}-1\times 10^{4}(R^{2}=0.992)$$

### 3.5. Experimental Design

A preliminary dose–response experiment was conducted to estimate the half-maximal effective concentration (EC_50_) of the SeNP preparation. Based on a previous study evaluating selenium toxicity in *Chlorella vulgaris* using concentrations of 25, 50, 75, 150, and 200 mg L^−1^ [68], we tested nominal concentrations of 0, 50, 100, 120, 150, 200, and 220 mg L^−1^. The inclusion of 100 and 120 mg L^−1^ provided greater resolution near the anticipated EC_50_, whereas the upper concentrations extended the exposure range sufficiently to capture marked growth inhibition.

For the main experiment, cultures of *Cladocopium* sp. and *Durusdinium* sp. were separately inoculated into 1 L conical flasks at an initial cell density of 5 × 10^5^ cells mL^−1^. For each species, four exposure conditions were established: a medium-only control and nominal mass concentrations of 50, 100, and 150 mg L^−1^ of the purified, freeze-dried SeNP preparation. The individual culture flask was considered the experimental unit.

Separate flasks were prepared for each species, treatment, and sampling time. At each sampling time of 24, 48, 72, and 96 h, three independent replicate flasks were used per treatment (*n* = 3). The designated flasks were destructively sampled and were not returned to the incubator or used at subsequent time points. Therefore, observations obtained at different sampling times originated from independent experimental units rather than repeated measurements of the same cultures, thereby avoiding temporal pseudo-replication.

For each flask, three aliquots were collected for parallel technical measurements. These technical replicate values were averaged to obtain a single biological replicate value for that flask and were not treated as independent observations. The independent flask groups collectively described the temporal response of each treatment over the 96 h exposure period. All cultures were maintained under identical conditions and were gently mixed three times daily to minimize cell aggregation and sedimentation.

### 3.6. Determination of Photosynthetic Pigments and Efficiency

Photosynthetic pigments were measured following Liang et al. [69]. Briefly, 10 mL of algal culture was centrifuged at 5000× *g* for 5 min. The pellet was extracted with 1 mL of 100% methanol, vortexed, and incubated in the dark at 4 °C for 24 h.

Absorbance was measured at 480, 510, 630, 664, and 750 nm using a Multiskan SkyHigh microplate reader (Thermo Fisher Scientific, Waltham, MA, USA). Pigment concentrations were calculated using Ritchie’s equations [67]:

$$Chlorophyll\;a\;\left(\mu g\;mL^{-1}\right)=13.6849\times \left(A_{664}-A_{750}\right)-3.4551\times \left(A_{630}-A_{750}\right)$$

$$Carotenoids\;\left(\mu g\;mL^{-1}\right)=7.6\times \left(A_{480}-A_{750}\right)-1.49\times \left(A_{510}-A_{750}\right)$$

Maximum photochemical efficiency of PSII (Fv/Fm) was determined after 30 min dark adaptation using a pulse-amplitude-modulated fluorometer (DUAL-PAM-100, Walz), the maximum fluorescence value (Fm) was determined by a 0.6 s saturation pulse (approximately 8000 μmol m^−2^·s^−1^) following Guo et al. [70].

### 3.7. Antioxidant Enzyme and Oxidative Stress Analysis

To evaluate oxidative stress responses, activities of antioxidant enzymes-superoxide dismutase (SOD), catalase (CAT), and peroxidase (POD)-were measured, along with levels of malondialdehyde (MDA) and hydrogen peroxide (H_2_O_2_).

Samples were collected at 24, 48, 72, and 96 h, with three replicates per treatment. All assays were conducted using commercial kits (Suzhou Greis Biotechnology Co., Ltd., Suzhou, China; catalog Nos. G0101W, G0105W, G0107W, G0109W, and G0168W, respectively) according to the manufacturer’s instructions. Prior to enzyme activity and biochemical assays, algal cells were harvested by centrifugation (7155× *g*, 10 min, 4 °C). After removal of the supernatant, the cell pellet was immediately weighed to determine fresh weight (FW). Enzyme activities and metabolite levels were normalized to protein content or cell density where appropriate.

### 3.8. Determination of Osmoregulatory Substances

To further elucidate the mechanisms underlying nano-Se effects, the contents of Osmo protectants, including soluble sugars (SS) and proline (PRO), were determined. Samples were collected at the same time points (24–96 h), and analyses were performed using commercial kits (Suzhou Greis Biotechnology Co., Ltd., Suzhou, China) following standardized protocols.

### 3.9. Statistical Analysis

Statistical analyses were performed using OriginPro 2026 (OriginLab Corporation, Northampton, MA, USA). The individual culture flask was treated as the experimental unit, and technical replicate measurements obtained from the same flask were averaged before analysis. Data are presented as the mean ± standard deviation (SD) of three independent biological replicates, with individual replicate values displayed in the figures. Differences among treatments were analyzed using one-way analysis of variance (ANOVA) followed by Tukey’s post hoc test, with *p* < 0.05 considered statistically significant. Pearson correlation analysis was performed separately for *Cladocopium* sp. and *Durusdinium* sp. to evaluate associations among the 12 measured physiological and biochemical variables. For each species, the three independent biological replicates within each concentration × time combination were averaged before analysis. The four nominal SeNP concentrations and four sampling times therefore provided 16 observations for each variable per species. Because the 12-variable matrix generated 66 pairwise correlations per species, the corresponding *p*-values were adjusted using the Benjamini–Hochberg false discovery rate procedure. Correlations were considered statistically significant at an adjusted q value < 0.05. Both unadjusted *p*-values and FDR-adjusted q-values were reported. Correlation coefficients were interpreted as statistical associations and not as evidence of causality.

## 4. Conclusions

This study demonstrates that green-synthesized nano-selenium exerts pronounced concentration-dependent and species-specific effects on the growth, photosynthesis, antioxidant defense, and osmotic regulation of symbiotic dinoflagellates. Both *Cladocopium* sp. and *Durusdinium* sp. exhibited a typical hormetic response pattern, with low concentrations promoting physiological performance and high concentrations inducing stress and growth inhibition. At low concentrations, nano-selenium enhanced photosynthetic pigment synthesis, improved PSII efficiency, and activated coordinated antioxidant and osmotic regulatory systems, thereby reducing oxidative damage and supporting cellular homeostasis. However, excessive nano-selenium disrupted redox balance, impaired photosynthetic function, and increased membrane lipid peroxidation, ultimately suppressing growth.

Notably, the two species adopted distinct adaptive strategies. *Cladocopium* sp. displayed a rapid-response strategy characterized by strong inducible increases in photosynthesis, antioxidant activity, and osmolyte accumulation, resulting in enhanced growth but lower tolerance to high-dose stress. In contrast, *Durusdinium* sp. exhibited a tolerance-oriented strategy, maintaining stable photosynthetic performance, balanced antioxidant activity, and higher baseline osmotic regulation, enabling greater resilience under elevated nano-selenium stress. Mechanistically, these findings indicate that nano-selenium functions as a redox-active nano modulator that regulates intracellular homeostasis through coordinated antioxidant and osmotic responses. This may improve the growth and stress resistance of symbiotic dinoflagellates. The identification of species-specific responses and concentration ranges provides a basis for the controlled application of nano-selenium in coral nursery systems. By modulating redox-dependent physiological processes in coral symbiotic dinoflagellates, nano-selenium may offer a promising and environmentally friendly strategy for improving coral health and nursery management.

## Data Availability

The original contributions presented in this study are included in the article. Further inquiries can be directed to the corresponding authors.

## References

[1] Wu D., Miao L., Song Y., Wang S., Wang T., Ou H., Xie J., Zhang Y., Jin C., Xia W. (2025). The distribution, diversity, and indicator species of coral communities under the influence of environmental changes in the subtropical peninsula of southern China. Ecol. Evol..

[2] Hagedorn M., Zuchowicz N., Henley E.M., Lager C., Perry R., Blackburn H., Bouwmeester J., Brunel O., Carter C., Rodriguez-Clark K.M. (2025). Conservation of coral genetic diversity through a global biorepository network. BioScience.

[3] Torres A.F., Valino D.A.M., Ravago-Gotanco R. (2021). Zooxanthellae Diversity and Coral-Symbiont Associations in the Philippine Archipelago: Specificity and adaptability across thermal gradients. Front. Mar. Sci..

[4] Sikorskaya T.V., Ginanova T.T., Ermolenko E.V., Boroda A.V. (2025). Lipidomic and physiological changes in the coral Acropora aspera during bleaching and recovery. Sci. Rep..

[5] Shabani F., Raie M., Kabiri K. (2025). Studying the impact of sea surface temperature (SST) changes and El Niño phenomenon on coral reefs bleaching around Kish Island in the northern Persian Gulf: A remote sensing approach. Deep. Sea Res. Part II Top. Stud. Oceanogr..

[6] Cavailles J., Kuzmics C., Grube M. (2025). Symbiont dynamics and coral regulation under changing temperatures. Coral Reefs.

[7] Roberty S., Plumier J.C. (2022). Bleaching physiology: Who’s the ‘weakest link’—Host vs. symbiont?. Emerg. Top. Life Sci..

[8] Yasir R.M., Zaki N.H. (2025). Antioxidant and anticancer activities of Bio-Synthesized selenium nanoparticles by *Escherichia coli*. Asian Pac. J. Cancer Prev. APJCP.

[9] Ding Q., Shi C., Wu H., Chen Y., Li W., Yang H., Zhu L., Ye L., Peng Y., Mei L. (2026). Selenium-derived bioactive enablers for advanced therapies: From molecular redox modulation to broad-spectrum disease applications. Bioact. Mater..

[10] Xue S.-J., Shu X., Xu T., Guo K.-X., Liu M.-L., Zhang J.-Y. (2026). The marine yeast Scheffersomyces spartinae 12SS-9 as a promising biological platform for robust biosynthesis of selenium nanoparticles with antioxidant and nutritional supplement potential. Food Biosci..

[11] Bohorquez J., Fine M., Grieco D.I., Gill D.A., Al-Sawalmih A., Padilla-Gamiño J., Bolden I.W., Hein M., Backstrom C., McField M. (2025). Coral reefs span borders, so must solutions: Transboundary conservation in complex political environments. npj Ocean. Sustain..

[12] Lin S., Wu S., He J., Wang X., Grossman A.R. (2024). Shining light on dinoflagellate photosystem I. Nat. Commun..

[13] Yang Q., Zhang H., Qiu J.-W., Huang D., Zhou X., Zheng X. (2025). Symbiotic Symbiodiniaceae mediate coral-associated bacterial communities along a natural thermal gradient. Environ. Microbiome.

[14] Guibert I. (2024). Assisted evolution of algal symbionts to enhance coral reef bleaching tolerance: A success story. Glob. Change Biol..

[15] De Mendoza A., Bonnet A., Vargas-Landin D.B., Ji N., Li H., Yang F., Li L., Hori K., Pflueger J., Buckberry S. (2018). Recurrent acquisition of cytosine methyltransferases into eukaryotic retrotransposons. Nat. Commun..

[16] Davies S.W., Gamache M.H., Howe-Kerr L.I., Kriefall N.G., Baker A.C., Banaszak A.T., Bay L.K., Bellantuono A.J., Bhattacharya D., Chan C.X. (2023). Building consensus around the assessment and interpretation of Symbiodiniaceae diversity. PeerJ.

[17] Pochon X., LaJeunesse T.C. (2021). Miliolidium n. gen, a New Symbiodiniacean Genus Whose Members Associate with Soritid Foraminifera or Are Free-Living. J. Eukaryot. Microbiol..

[18] Matos A., Antunes A. (2021). Symbiotic associations in ascidians: Relevance for functional innovation and bioactive potential. Mar. Drugs.

[19] Nelson H.R., Altieri A.H. (2019). Oxygen: The universal currency on coral reefs. Coral Reefs.

[20] Li J., Li J., Shao Z., Cheng K., Yang Q., Ju H., Tang X., Zhang S., Li J., Li J. (2025). Coral-associated Symbiodiniaceae exhibit host specificity but lack phylosymbiosis, with *Cladocopium* and *Durusdinium* showing different cophylogenetic patterns. New Phytol..

[21] Sawiccy V., Tjandra N.W., Maruyama S., Ruggeri M., Vo C., Harmon L.A., Poole A.Z., Weis V.M. (2025). Regulatory role of NADPH oxidases in symbiosis and dysbiosis in the sea anemone Aiptasia. Front. Mar. Sci..

[22] Majerová E., Steinle C., Drury C. (2025). BAK knockdown delays bleaching and alleviates oxidative DNA damage in a reef-building coral. Commun. Biol..

[23] Karp R.F., Lepiz-Conejo F., Matsuda S.B., Corbett B., Wen A.D., Unsworth J.D., D’Alessandro M., Nedimyer K., Moura A., Muller E.M. (2025). Heat-tolerant algal symbionts may prevent extirpation of the threatened elkhorn coral, Acropora palmata, in Florida during intensifying marine heatwaves. Coral Reefs.

[24] Zhang J., Kang W., Downs C.A., Zhou Y., Zhou Z., Tang Z., Zhao H., Chou L.-M., Wu F. (2026). Nitrate Aggravates While Ammonium Mitigates Thermal Bleaching in Corals through Divergent Lipid-Mediated Pathways and Stress Response. Environ. Sci. Technol..

[25] Pogoreutz C., Oakley C.A., Rädecker N., Cárdenas A., Perna G., Xiang N., Peng L., Davy S.K., Ngugi D.K., Voolstra C.R. (2022). Coral holobiont cues prime Endozoicomonas for a symbiotic lifestyle. ISME J..

[26] Gong S., Liang J., Li G., Xu L., Tan Y., Zheng X., Jin X., Yu K., Xia X. (2023). Linking coral fluorescence phenotypes to thermal bleaching in the reef-building Galaxea fascicularis from the northern South China Sea. Mar. Life Sci. Technol..

[27] Botanical Society of Edinburgh (1897). Transactions and proceedings of the Botanical Society of Edinburgh. Trans. Bot. Soc. Edinb..

[28] Turnham K.E., Wham D.C., Sampayo E., LaJeunesse T.C. (2021). Mutualistic microalgae co-diversify with reef corals that acquire symbionts during egg development. ISME J..

[29] McIlroy S.E., Cunning R., Baker A.C., Coffroth M.A. (2019). Competition and succession among coral endosymbionts. Ecol. Evol..

[30] Wang C., Zheng X., Li Y., Sun D., Huang W., Shi T. (2022). Symbiont shuffling dynamics associated with photodamage during temperature stress in coral symbiosis. Ecol. Indic..

[31] Chen Y., Liu W., Leng X., Stoll S. (2021). Toxicity of selenium nanoparticles on Poterioochromonas malhamensis algae in Waris-H culture medium and Lake Geneva water: Effect of nanoparticle coating, dissolution, and aggregation. Sci. Total Environ..

[32] Suggett D.J., Warner M.E., Leggat W. (2017). Symbiotic Dinoflagellate Functional Diversity Mediates Coral Survival under Ecological Crisis. Trends Ecol. Evol..

[33] Özkaya D., Nazıroğlu M., Vanyorek L., Muhamad S. (2021). Involvement of TRPM2 channel on Hypoxia-Induced oxidative injury, inflammation, and cell death in retinal pigment epithelial cells: Modulator action of selenium nanoparticles. Biol. Trace Elem. Res..

[34] Sidhu A.K., Verma N., Kaushal P. (2022). Role of biogenic capping agents in the synthesis of metallic nanoparticles and evaluation of their therapeutic potential. Front. Nanotechnol..

[35] Ma Y., Yuan W., Kan W., Huang C., Zhu J., Zhang G., Li H., Balamurugan S., Wu L. (2022). Quercetin rapidly potentiates the biogenesis of nanoselenium via orchestrating key signaling pathways in Chlorella vulgaris. Chem. Eng. J..

[36] Dawood M.A.O., Basuini M.F.E., Yilmaz S., Abdel-Latif H.M.R., Kari Z.A., Razab M.K.A.A., Ahmed H.A., Alagawany M., Gewaily M.S. (2021). Selenium nanoparticles as a natural antioxidant and metabolic regulator in aquaculture: A review. Antioxidants.

[37] Peng S.-Y., Yan J., Li M., Yan Z.-X., Wei H.-Y., Xu D.-J., Cheng X. (2023). Preparation of polysaccharide-conjugated selenium nanoparticles from spent mushroom substrates and their growth-promoting effect on rice seedlings. Int. J. Biol. Macromol..

[38] Umapathy S., Pan I. (2025). Glucose reduced nano-Se mitigates Cu-induced ROS by upregulating antioxidant genes in zebrafish larvae. Nanoscale Adv..

[39] Bell S.L., Quigley K.M. (2025). Global Free-Living Symbiodiniaceae Biodiversity Mirrors Local Environments. J. Biogeogr..

[40] Dubé C.E., Hume B.C.C., Boissin E., Mercière A., Bourmaud C.A.-F., Ziegler M., Voolstra C.R. (2023). Algal symbioses with fire corals demonstrate host genotype specificity and niche adaptation at subspecies resolution. BioRxiv.

[41] Ahmed Z.M., Ahmed M.E. (2025). Physicochemical analysis of selenium nanoparticles synthesis via pomegranate peel extracts approach: Cytotoxic impact on MCF7 cell line. Iraqi J. Chem. Pet. Eng..

[42] Ghaderi R.S., Adibian F., Sabouri Z., Davoodi J., Kazemi M., Jamehdar S.A., Meshkat Z., Soleimanpour S., Daroudi M. (2021). Green synthesis of selenium nanoparticle by Abelmoschus esculentus extract and assessment of its antibacterial activity. Mater. Technol..

[43] Ahmed M.E., Sulaiman G.M., Hasoon B.A., Khan R.A., Mohammed H.A. (2024). Green Synthesis and Characterization of Apple Peel-Derived Selenium Nanoparticles for Anti-Fungal Activity and Effects of MexA Gene Expression on Efflux Pumps in Acinetobacter baumannii. Appl. Organomet. Chem..

[44] Prakash J.M., Pethappachetty P., Kannurkaran R., Valiyaparambil S., Kolli D. (2025). Bioengineered selenium nanoparticles synthesized using Ficus hispida ethyl acetate extract exhibit antibacterial and antioxidant activities. Microb. Pathog..

[45] Barzegarparay F., Najafzadehvarzi H., Pourbagher R., Parsian H., Ghoreishi S.M., Mortazavi-Derazkola S. (2023). Green synthesis of novel selenium nanoparticles using Crataegus monogyna extract (SeNPs@CM) and investigation of its toxicity, antioxidant capacity, and anticancer activity against MCF-7 as a breast cancer cell line. Biomass Convers. Biorefinery.

[46] Wang S., Zhang N., Xu H., Tan L., Wang J. (2024). Allelochemicals of Alexandrium tamarense and its algicidal mechanism for Prorocentrum donghaiense and Heterosigma akashiwo. Chemosphere.

[47] Liu S., Han J., Yao L., Li H., Xin G., Ho S.-H., Huang X. (2024). Integrated multilevel investigation of photosynthesis revealed the algal response distinction to differentially charged nanoplastics. J. Hazard. Mater..

[48] Agathokleous E., Feng Z., Iavicoli I., Calabrese E.J. (2019). The two faces of nanomaterials: A quantification of hormesis in algae and plants. Environ. Int..

[49] Shoman N., Solomonova E., Akimov A., Rylkova O., Mansurova I. (2024). Activation of stress reactions in the dinophyte microalga Prorocentrum cordatum as a consequence of the toxic effect of ZnO nanoparticles and zinc sulfate. Aquat. Toxicol..

[50] Chen H., He Y., Liang M., Yan B., Jiang J. (2021). The expression pattern of β-carotene ketolase gene restricts the accumulation of astaxanthin in Dunaliella under salt stress. J. Cell. Physiol..

[51] Liu S., Huang X., Han J., Yao L., Li H., Xin G., Ho S.-H., Zhao J., Xing B. (2024). Genome-wide molecular adaptation in algal primary productivity induced by prolonged exposure to environmentally realistic concentration of nanoplastics. ACS Nano.

[52] Roberty S., Furla P., Plumier J.-C. (2016). Differential antioxidant response between two Symbiodinium species from contrasting environments. Plant Cell Environ..

[53] Huang Y., Gao B., Long C., Wang Y., Long L., Yang F. (2024). The growth and nitrogen utilization strategies in two dominant Symbiodiniaceae species facing nitrogen deficiency and enrichment. Algal Res..

[54] McIlroy S.E., Wong J.C.Y., Baker D.M. (2020). Competitive traits of coral symbionts may alter the structure and function of the microbiome. ISME J..

[55] Poquita-Du R.C., Huang D., Chou L.M., Todd P.A. (2020). The contribution of stress-tolerant endosymbiotic dinoflagellate *Durusdinium* to Pocillopora acuta survival in a highly urbanized reef system. Coral Reefs.

[56] Zhang H., Gu B., Zhou Y., Ma X., Liu T., Xu H., Xie Z., Liu K., Wang D., Xia X. (2022). Multi-Omics profiling reveals resource allocation and acclimation strategies to temperature changes in a marine dinoflagellate. Appl. Environ. Microbiol..

[57] AliBabazadeh B., Rahmani F., Agh N. (2025). Multifaceted stress response in Tetradesmus obliquus to selenium dioxide exposure: Antioxidant defense and metabolic adaptations. Environ. Sci. Pollut. Res..

[58] Tortorelli G., Rosset S.L., Sullivan C.E.S., Woo S., Johnston E.C., Walker N.S., Hancock J.R., Caruso C., Varela A.C., Hughes K. (2025). Heat-induced stress modulates cell surface glycans and membrane lipids of coral symbionts. ISME J..

[59] Song J., Xu Y., Liu C., He Q., Huang R., Jiang S., Ma J., Wu Z., Huangfu X. (2020). Interpreting the role of NO^3−^, SO_4_^2−^, and extracellular polymeric substances on aggregation kinetics of CeO_2_ nanoparticles: Measurement and modeling. Ecotoxicol. Environ. Saf..

[60] Chen W., Cheng H., Xia W. (2022). Construction of Polygonatum sibiricum Polysaccharide Functionalized Selenium Nanoparticles for the Enhancement of Stability and Antioxidant Activity. Antioxidants.

[61] Coley P.D., Bryant J.P., Chapin F.S. (1985). Resource availability and plant antiherbivore defense. Science.

[62] Singh S., Singh P., Singh A., Singh S., Prasad N., Asthana R.K. (2025). Deciphering selenium tolerance in a halotolerant microalga Dunaliella salina. Plant Stress.

[63] Lee J.-W., Lee M.-W., Nam K.-H., Chun S.-J., Oh H.-M., Jin E., Lee H.-G. (2024). Enhancement of ketocarotenoid production via heterologous expression of orange protein from Ipomoea batatas in indigenous microalga *Ettlia* sp.. Algal Res..

[64] Li M., Jiang Y., Chuang C.-Y., Zhou J., Zhu X., Chen D. (2019). Recovery of Alexandrium tamarense under chronic exposure of TiO_2_ nanoparticles and possible mechanisms. Aquat. Toxicol..

[65] Borowska M., Pszczoła J., Pawlak K., Ruzik L., Ombugadu J.N., Trzaskowski M., Jankowski K. (2025). Microwave-assisted green synthesis of selenium nanoparticles using citrus extracts: Insights into size-controlled formation and surface characteristics. Colloids Surf. A Physicochem. Eng. Asp..

[66] Saravanan K., Madhaiyan M., Periyasamy P., Manivannan P., Bayrakdar A., Balakrishnan V. (2025). Green synthesis and detailed characterization of selenium nanoparticles derived from *Alangium salviifolium* (L.f) Wangerin. Chem. Phys. Impact.

[67] Ritchie R.J. (2006). Consistent sets of spectrophotometric chlorophyll equations for acetone, methanol and ethanol solvents. Photosynth. Res..

[68] Sun X., Zhong Y., Huang Z., Yang Y. (2014). Selenium Accumulation in Unicellular Green Alga Chlorella vulgaris and Its Effects on Antioxidant Enzymes and Content of Photosynthetic Pigments. PLoS ONE.

[69] Liang J., Qin L., Zhang L., Xu Y., Niu T., Li Z., Yang Y., Liang Z., Yu K., Gong S. (2025). The stress response strategies of two typical coral Symbiodiniaceae (*Cladocopium goreaui* and *Durusdinium trenchii*) under abnormal temperatures. Algal Res..

[70] Guo Y.P., Guo D.P., Zhou H.F., Hu M.J., Shen Y.G. (2006). Photoinhibition and xanthophyll cycle activity in bayberry (*Myrica rubra*) leaves induced by high irradiance. Photosynthetica.

